# Overcoming borders: International cooperation in re-use and re-interpretation of omics data in Fontan circulation^☆^

**DOI:** 10.1016/j.ijcchd.2025.100590

**Published:** 2025-05-05

**Authors:** Miriam Michel, David Renaud, Benjamin Kelly, Ismael Z. Assi, Liming Pei, Alexander R. Opotowsky

**Affiliations:** a Department of Child and Adolescent Health, Division of Pediatrics III—Cardiology, Pulmonology, Allergology and Cystic Fibrosis, Medical University of Innsbruck, 6020, Innsbruck, Austria; b Department of Cardiothoracic Surgery, Copenhagen University Hospital-Rigshospitalet, Copenhagen, Denmark; c Heart Institute, Department of Pediatrics, Cincinnati Children's Hospital and University of Cincinnati College of Medicine, Cincinnati, OH, USA; d University of Cincinnati College of Medicine, Cincinnati, OH, USA; e Center for Mitochondrial and Epigenomic Medicine, Cardiovascular Institute, Department of Pathology and Laboratory Medicine, Children's Hospital of Philadelphia, and Perelman School of Medicine, University of Pennsylvania, Philadelphia, PA, USA

**Keywords:** Data re-use, Fontan, Omics, Single ventricle

Dear editor,

Development and application of omics technologies are evolving rapidly. Data sharing via public repositories is becoming the standard in research involving large volumes of data, not only rendering scientific approaches repeatable but also allowing for re-use of data. The re-use and aggregation of shared datasets is especially valuable in research on orphan diseases with only small heterogeneous patient groups, such as complex congenital heart disease palliated by Fontan surgery [1].

Within the past five years a number of omics studies have been published on the Fontan circulation [[2], [3], [4], [5], [6], [7], [8]]. Most of them followed an exploratory approach to characterize the Fontan circulation, revealing marked alterations of metabolic profiles suggesting globally altered regulatory processes with respect to endothelial structure and function, inflammation, oxidative stress, immune system dysregulation, and an energy deficient state present in patients both with and without overt complications.

These studies have provided novel insights, generating a plethora of hypotheses. But there are several limitations. First, the small number of patients included. Second, the large phenotypic heterogeneity both in Fontan-specific complications, and with respect to ‘basic’ key factors determining outcomes such as the morphology of the systemic ventricle, or functional phenotype (e.g., exercise capacity).

For these reasons, systematic comparison of results, such as summarizing data in a meta-analysis, is challenging. Reducing the complex situation of a Fontan circulation to single key factors like congestion or long-standing hypoxemia is tempting. However, the informative value of those simplified approaches is limited, depending on initial assumptions [9].

One complication common with the Fontan circulation is Fontan-associated liver disease (FALD). Though histomorphological features of FALD have been well characterized [10], the underlying molecular and metabolic features, particularly the role of inflammation in fibrogenesis, are not entirely understood. Early detection of FALD is challenging primarily due to the nature of Fontan hemodynamics, sustained hepatic function (esp. protein synthesis), and frequently normal levels of transaminases, limiting the informative value of blood testing, imaging, and elastography.

Research groups from Northern American centers have leveraged routine clinical liver biopsy protocols to directly assess biological processes using existing liver tissue. In 2024, two landmark omics studies were published, focusing on the transcriptome of liver tissue of patients with histomorphologically defined FALD.

First, Bravo-Jaimes et al. [11] performed bulk RNA sequencing in over 100 patients. The investigators reported upregulation of genes important in inflammation, VEGFA-VEGFR2 and TGF-β signaling pathways, and vasculature development in advanced fibrotic livers compared to early fibrosis. The inflammation pathway in advanced fibrotic stage is particularly interesting as it is not generally recognized as a key histomorphological feature in FALD. Their findings provided important biological insights into FALD. However, the authors did not compare their findings to tissue samples of healthy or patients with other forms of liver disease and did not indicate patient age at biopsy, a critical predictor of FALD severity.

Second, Hu et al. [12] published single-cell multiomics (gene expression and chromatin accessibility from the same liver cells) analysis of livers from a small group of adolescent Fontan patients and controls. They focused on adolescent age aiming to identify early and likely causal pathogenetic mechanisms of FALD. The results revealed profound cell type–specific transcriptomic and epigenomic changes in FALD. Importantly, central hepatocytes were found to exhibit the most substantial changes, featuring significant metabolic reprogramming. These central hepatocytes changes preceded substantial activation of hepatic stellate cells and liver fibrosis, suggesting central hepatocytes as the potential first “responder” in the pathogenesis of FALD. They also discovered signals from central hepatocytes to hepatic stellate cells that may promote their activation and liver fibrosis. One intriguing discovery is that in central hepatocytes bile acid metabolism gene expression is altered, opening discussion of the role of mild cholestasis and circulating blood biomarkers in the Fontan circulation [13,14].

In a follow up study, the same group performed untargeted liver metabolomics in 12 controls and 12 adolescent patients. The results confirmed altered bile acid metabolism, among others, and found disrupted amino acid metabolism as a key feature of FALD. Intriguingly, abnormal liver amino acid metabolism was shown to be one of the two common features among FALD and other common human liver diseases (MASH and MAFLD) [15].

Both raw and processed single-cell multiomics (Hu et al. [12]) and metabolomics (Sule et al. [15]) data were deposited in public repositories. This allowed Angelotti et al. [16] to analyze the single-cell multiomics data with a different focus. In this creative, efficient use of existing data, the authors focused on immune-function gene-regulation and inflammatory-cell subtypes, in particular the regulation of T-cells found in hepatic tissue. In so doing, Angelotti et al. set another key tone, emphasizing the potential importance of immunological processes in FALD.

Angelotti's approach not only provides a window into additional features of FALD pathophysiology, but also heralds a new era of multicenter collaboration by data re-use and hypothesis-driven reexamination of existing data. This is already common in many medical fields. We applaud efforts to perform focused, explanatory data re-analysis overcoming data sharing regulations while maintaining substantive human research protections, to advance collaborative science on the Fontan circulation and other congenital heart diseases [17,18].

## CRediT authorship contribution statement

**Miriam Michel:** Conceptualization, Funding acquisition, Supervision, Writing – original draft, Writing – review & editing, Project administration. **David Renaud:** Conceptualization, Supervision, Writing – original draft, Writing – review & editing. **Benjamin Kelly:** Writing – review & editing. **Ismael Z. Assi:** Writing – review & editing. **Liming Pei:** Writing – review & editing. **Alexander R. Opotowsky:** Supervision, Writing – review & editing.

## Disclosure

Alexander Opotowsky is an Editorial Board Member of the International Journal of Cardiology Congenital Heart Disease but played no role in the Journal's evaluation of the manuscript.

## Funding

This research was funded in whole or in part by the Austrian Science Fund (FWF) [grant no. KLI-1036B, grant-DOI 10.55776/KLI1036]. For open access purposes, the author has applied a CC BY public copyright license to any author-accepted manuscript version arising from this submission.

## Declaration of competing interest

The authors declare the following financial interests/personal relationships which may be considered as potential competing interests:Miriam Michel reports article publishing charges was provided by Austrian Science Fund. Miriam Michel reports a relationship with Austrian Science Fund that includes: funding grants. If there are other authors, they declare that they have no known competing financial interests or personal relationships that could have appeared to influence the work reported in this paper.
